# Supplementary material for: Insights into real-time chemical processes in a calcium sensor protein-directed dynamic library
Source: Nat Commun. 2019 Jun 26;10:2798. doi: 10.1038/s41467-019-10627-w (PMC6595003; doi:10.1038/s41467-019-10627-w)
Supplement: Supplementary file 1 — Supplementary Information [file 41467_2019_10627_MOESM1_ESM.pdf]

SUPPLEMENTARY INFORMATION

Insights into Real-Time Chemical Processes in a Calcium Sensor Protein-Directed Dynamic Library

Andrea Canal-Martín,[1,2] Javier Sastre,[1] María José Sanchez-Barrena,[3*] Angeles Canales,[2] Sara Baldominos,[1] Naiara Pascual,[1] Loreto Martínez-González,[1] Dolores Molero,[4] Mª Encarnación Fernández-Valle,[4] Elena Sáez,[4] Patricia Blanco-Gabella,[3] Elena Gómez-Rubio,[1] Sonsoles Martín-Santamaría,[1] Almudena Sáiz,[5] Alicia Mansilla,[5*] F. Javier Cañada,[1] Jesús Jiménez-Barbero,[6] Ana Martínez,[1] Ruth Pérez-Fernández[1*]

[1]Chemical and physical biology department, Centro de Investigaciones Biológicas, CIB-CSIC, Madrid 28040, Spain; [2]Organic chemistry department, Universidad Complutense de Madrid, Madrid 28040, Spain; [3]Department of Crystallography and Structural Biology, Instituto de Química Física Rocasolano, IQFR-CSIC, Madrid 28006, Spain; [4]CAI de RMN, Universidad Complutense de Madrid, 28040 Madrid, Spain; [5]Instituto Ramón y Cajal de Investigación Sanitaria. Ctra. Colmenar Viejo, km. 9100 28034 Madrid, Spain; [6]Molecular recognition and host-pathogen interactions, CIC bioGUNE, Derio 48160, Bizkaia, Spain.

ruth.perez@csic.es, xmjose@iqfr.csic.es, alicia.mansilla@salud.madrid.org

INDEX

1. **SUPPLEMENTARY FIGURES 2**

2. **SUPPLEMENTARY TABLES 11**

3. **SUPPLEMENTARY METHODS 17**

4. **SUPPLEMENTARY DISCUSSION 22**

5. **SUPPLEMENTARY REFERENCES 25**

1. SUPPLEMENTARY FIGURES

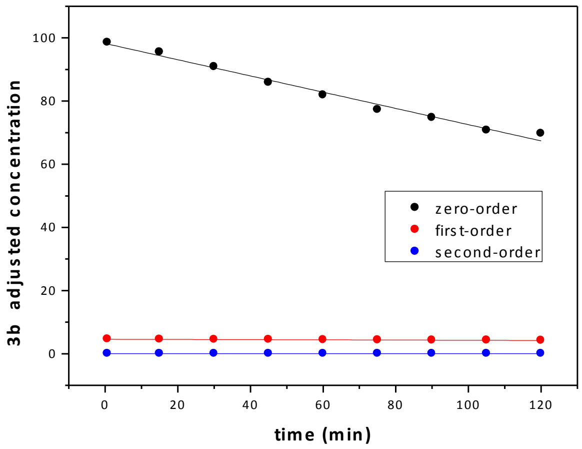

Supplementary Figure 1. Reaction order fittings. Black line is zero-order fitting [1] (μM) = -0.26 ± 0.01 · time (min) + 98.2 ± 0.8 , r^2 = 0.988 , red line is first-order fitting ln[1] = -0.0031 ± 0.0001 · time (min) + 4.595 ± 0.007, r^2 = 0.990, and blue line is second-order fitting $[1]^{-1}$ (μM^{-1}) = (3.8 ± 0.1)·10^{-5} · time (min) + (9.00 ± 0.08)·10^{-3}, r^2 = 0.994. Source data are provided as a Source Data file.

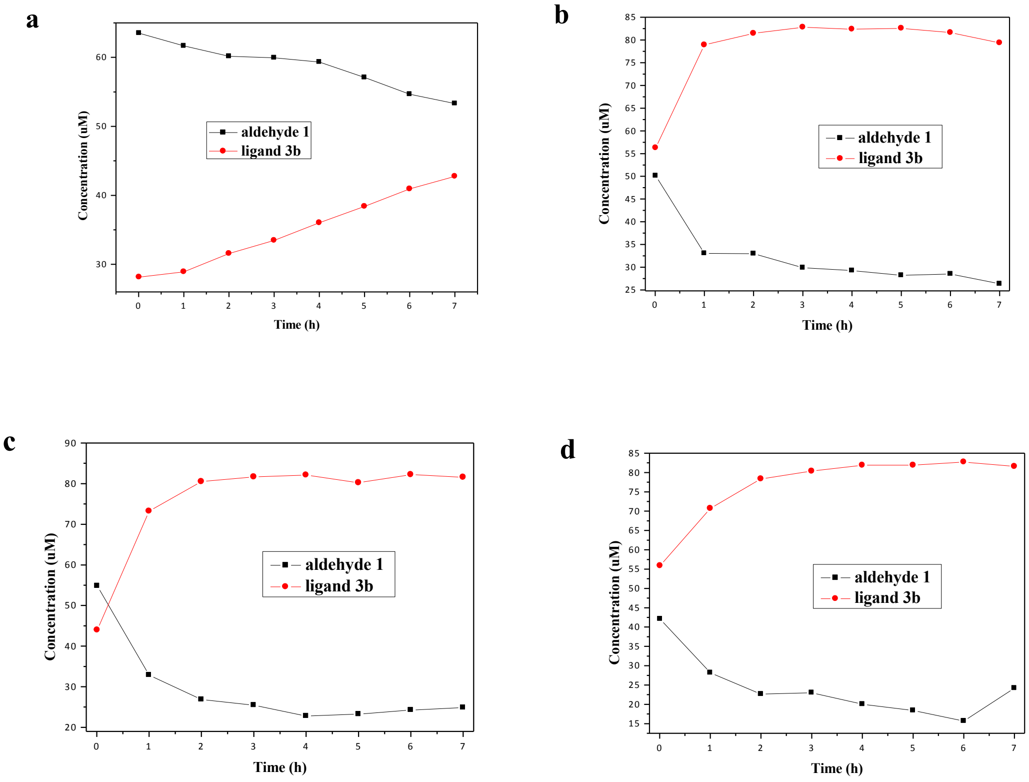

Supplementary Figure 2. Analysis of the reaction evolution for 7 hours. The aldehyde 1 (black dots) and the acylhydrazone 3b (red dots): **a,** in absence of catalyst; **b,** in presence of *p*-anisidine; **c,** in presence of *p*-phenylendiamine; **d,** in presence of aniline. Using 15 mM or 50 mM, the reaction course in 7 hours is similar. Source data are provided as a Source Data file.

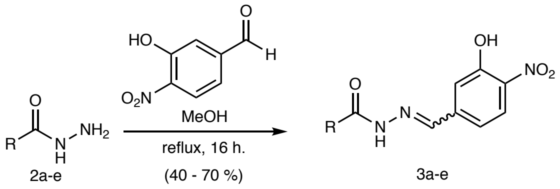

Supplementary Figure 3. General procedure of acylhydrazone reaction.

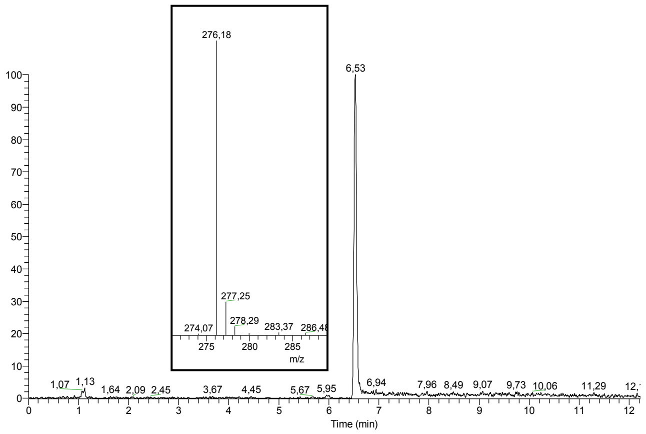

Supplementary Figure 4. HPLC and MS of acylhydrazone 3a.

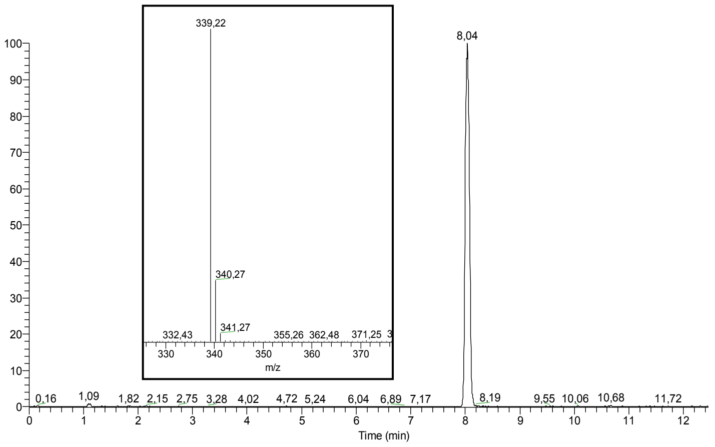

Supplementary Figure 5. HPLC and MS of acylhydrazone 3b.

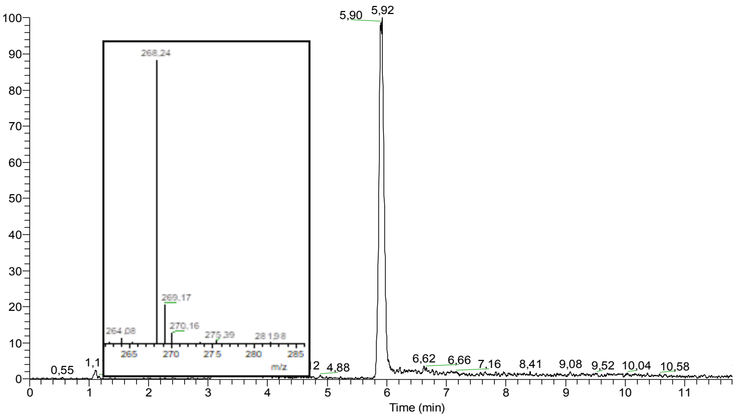

Supplementary Figure 6. HPLC and MS of acylhydrazone 3c.

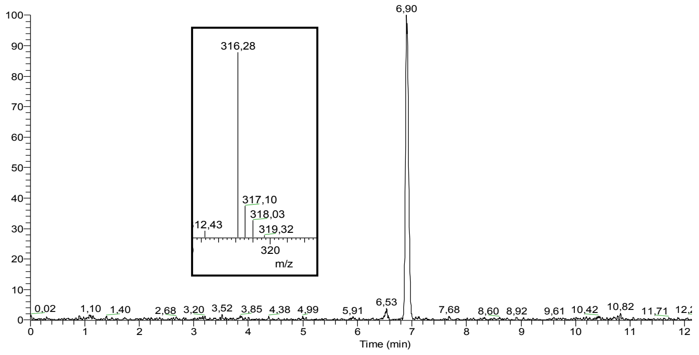

Supplementary Figure 7. HPLC and MS of acylhydrazone 3d.

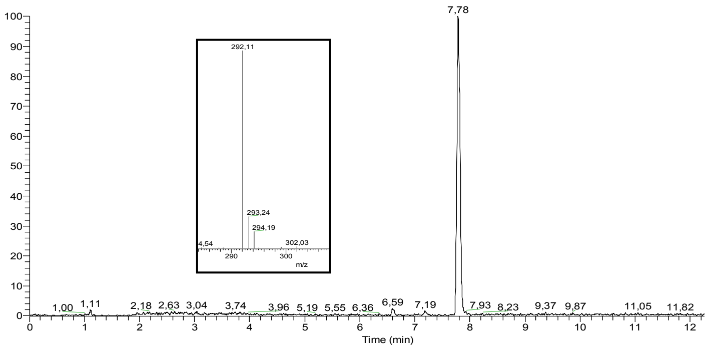

Supplementary Figure 8. HPLC and MS of acylhydrazone 3e.

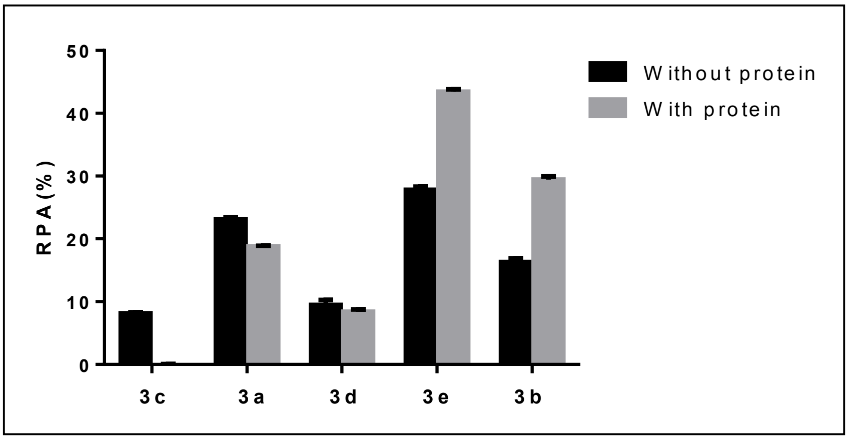

Supplementary Figure 9. Relative Peak Area of the DCL with and without *d*NCS1. Aldehyde 1 (1.2 µL, 50 mM), 2a-e (3.6 µL, 50 mM), catalyst (1 µL, 12 M), *d*NCS-1:1 [1:1], tris buffer (20 mM, pH 7.4), 1 mM $CaCl_2$, 0.5 M NaCl, 1 mM DTT, T = 4 ºC, 2% DMSO. Mean relative peak area (RPA) of each acylhydrazone of the library in the absence (blank) and presence of *d*NCS-1. Error bars indicate standard deviation (SD) over three experiments. The calculation is set by the sum of all peak areas to 100% and assigning each peak its percentage. Source data are provided as a Source Data file.

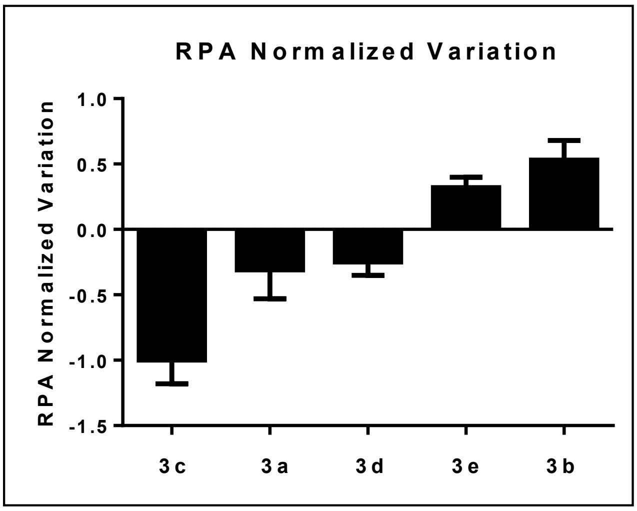

Supplementary Figure 10. RPA normalized of variation of DCL with and without *d*NCS1. Aldehyde 1 (1.2 µL, 50 mM), 2a-e (3.6 µL, 50 mM), catalyst (1 µL, 12 M), *d*NCS-1:1 [1:1], tris buffer (20 mM, pH 7.4), 1 mM $CaCl_2$, 0.5 M NaCl, 1 mM DTT, T = 4 ºC, 2% DMSO. Error bars indicate SD over three experiments. Source data are provided as a Source Data file.

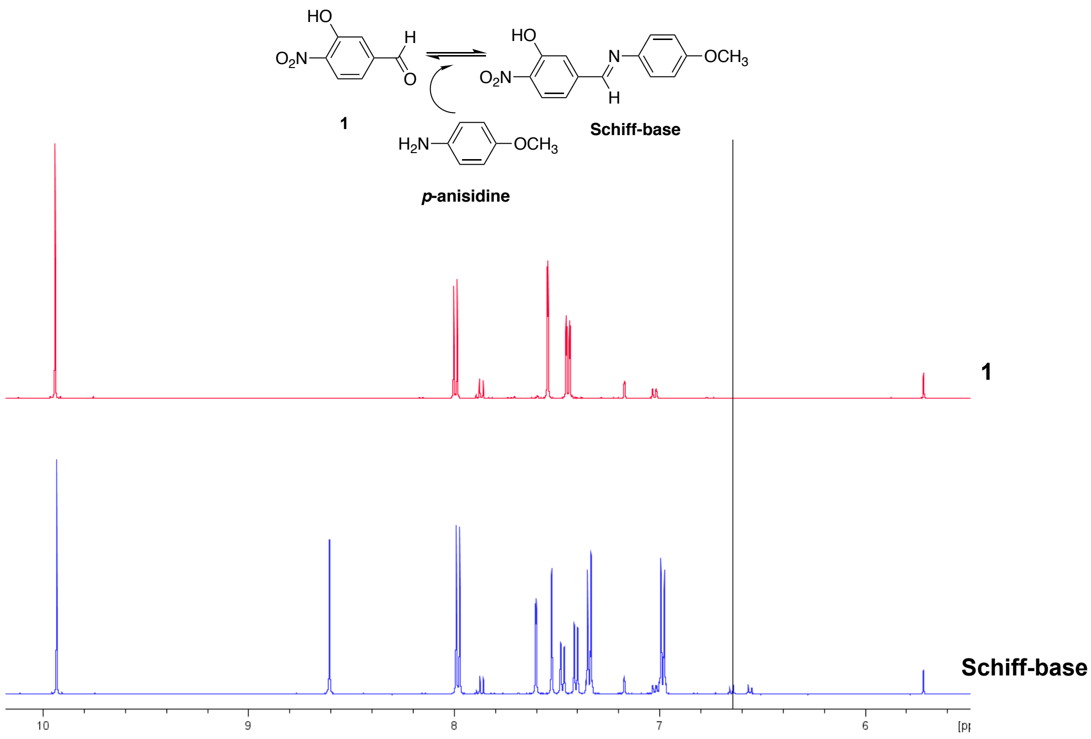

Supplementary Figure 11. Monodimensional ^{1}H-NMR of the aldehyde 1 and the Schiff base. Note that there is a small amount of the hydrate aldehyde present in the sample (t=0).

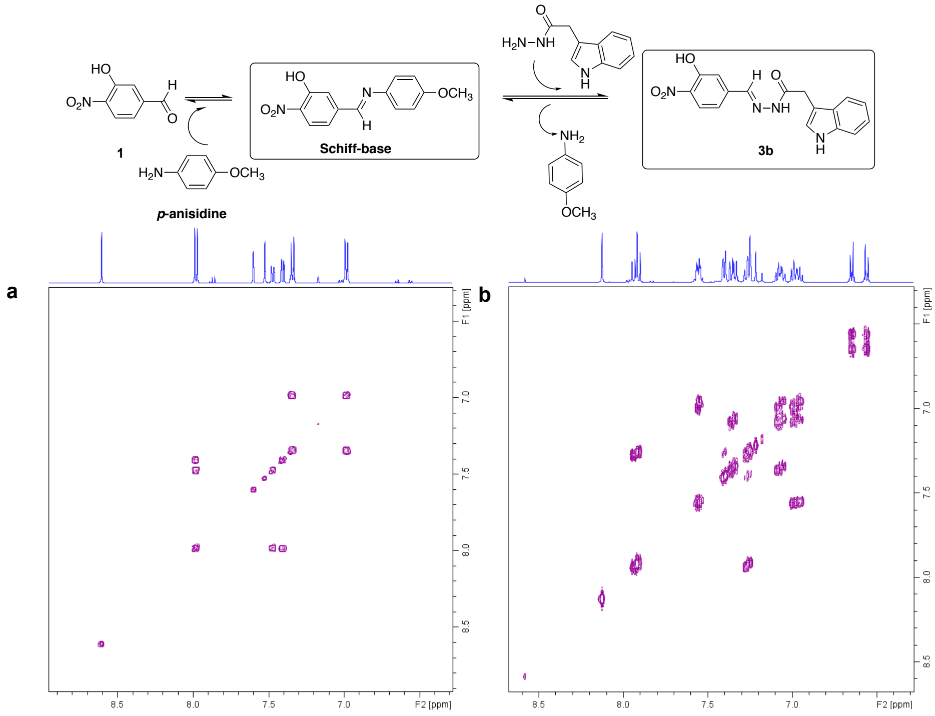

Supplementary Figure 12. TOCSY pictures. **a**, Schiff-base and **b**, the final compound 3b equilibrium. Starting and final state of the video recorded.

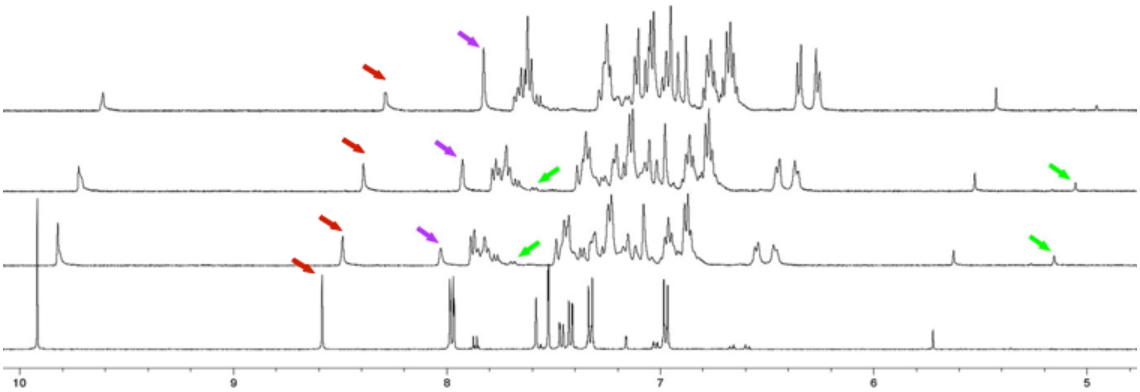

Supplementary Figure 13. ^{1}H-NMR monodimensional spectra. Reaction compounds 1, *p*-anisidine and 2b. Identification of the Intermediate I in the catalytic pathway (green arrows), protons belonging to the Schiff-base are highlighted in red and of compound 3b in purple.

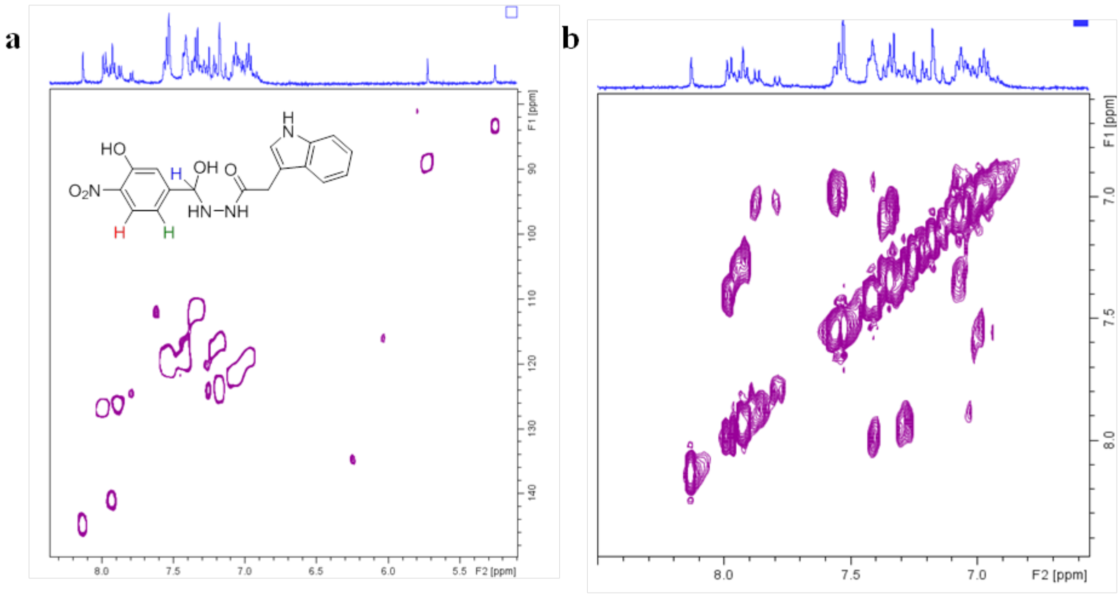

Supplementary Figure 14. Elucidation of Intermediate I structure. **a**, COSY NMR experiment of Intermediate I. **b**, COSY NMR spectra amplification (6-9 ppm).

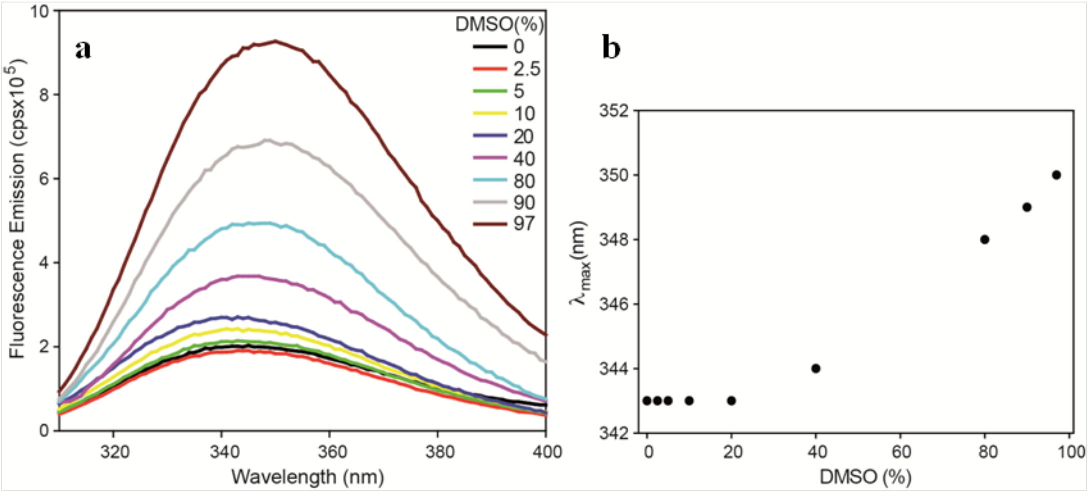

Supplementary Figure 15. *d*NCS1 tolerance to DMSO. **a,** Trp emission spectra at increasing concentrations of DMSO. **b,** representation of the emission maximum (λ_{max}) observed at each DMSO concentration. Source data are provided as a Source Data file.

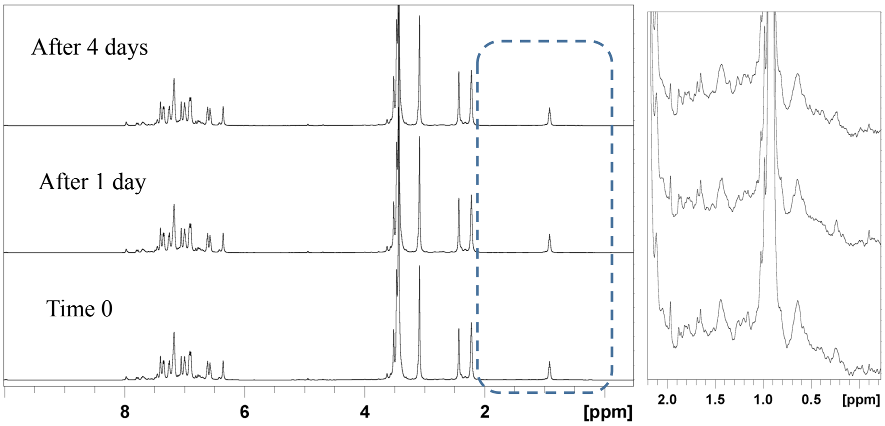

Supplementary Figure 16. DCL and protein spectra recorded at different times. 100:1 equivalents DCL:protein spectra was recorded at initial time of mixture, after one and after four days. Zoom on the area where signal of aliphatic side chain residues of the protein appear. Although the base line is very noisy, the profile of the protein signals does not change significantly. The spectra were acquired with 32 scans and processed with 8Hz line broadening for smoothing the noisy base line.

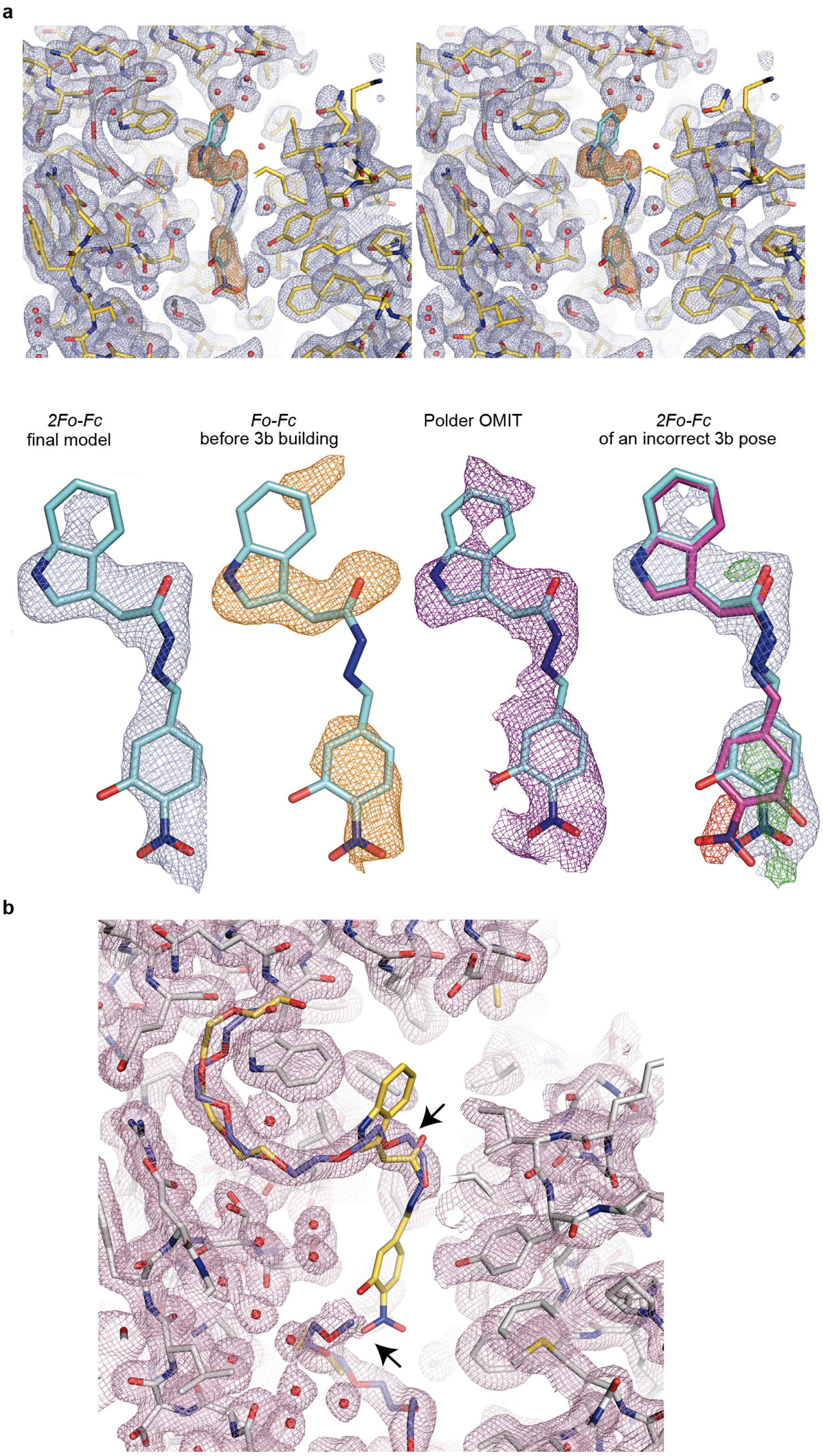

Supplementary Figure 17. Electron density map calculations at the ligand binding site. **a,** Stereo image of a portion of the $2F_o$-F_c electron density map (blue colour, 0.7σ) of the final *h*NCS-1/3b model. The protein, 3b and PEG molecules are depicted as sticks in yellow, cyan and light grey, respectively. Water molecules are displayed as red spheres. A F_o-F_c difference map (orange colour, 2.5σ) from the first cycles of refinement and before the ligand was built is

superposed. To visualize the fit of the structure to the different calculated maps, a zoom on the small molecule is shown. In addition, the Polder OMIT map is displayed in purple and 3.4σ. The Polder map correlation coefficients are CC(1,2): 0.7387, CC(1,3): 0.8152 and CC(2,3): 0.6739. Finally, to demonstrate that the 3b hydroxyl group is pointing to the solvent (final modelled pose, cyan) and not to the hydrophobic crevice (magenta), the conformation was modelled and refined (see right-hand side zoom). The corresponding $2F_o$-F_c (blue, 0.7σ) and F_o-F_c difference (red -3.0σ, green 3.0 σ) maps were calculated. The refined conformation, does not fit well in the resulting $2F_o$-F_c map. Also, the difference maps suggest changes that support the final modelled pose. **b,** The $2F_o$-F_c electron density map (pink colour, 0.7σ) of the native *h*NCS-1 structure (1g8i)[1] showing the region where 3b binds. The protein, and PEG molecules are depicted as sticks in light grey and lilac, respectively. The NCS-1/3b structure has been superimposed but only 3b and the surrounding PEG molecules are shown in yellow. The image illustrates that during the soaking of native *h*NCS-1 crystals with 3b, the ligand displaces PEG molecules for proper recognition. This, together with the moderate affinity that 3b shows for the protein, likely contributes to the partial occupancy of the ligand, which was refined to 0.73.

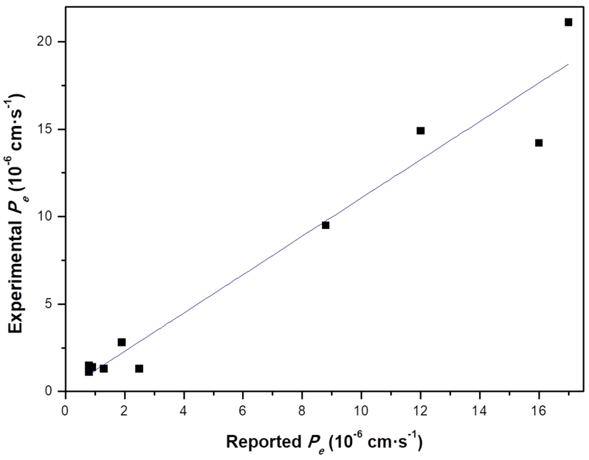

Supplementary Figure 18. Linear correlation between experimental and reported permeability. Linear correlation of permeabilities from commercial drugs using the PAMPA-BBB assay. A good correlation between experimental-described values was obtained P_e (exp.) = 1.094 (bibl.) + 0.1264 (R^2= 0.9531). From this equation and following the pattern established in the literature for BBB permeation prediction[2] we could classify compounds as CNS + when they present a permeability > 4.50 x 10^{-6} cm s^{-1}. Source data are provided as a Source Data file.

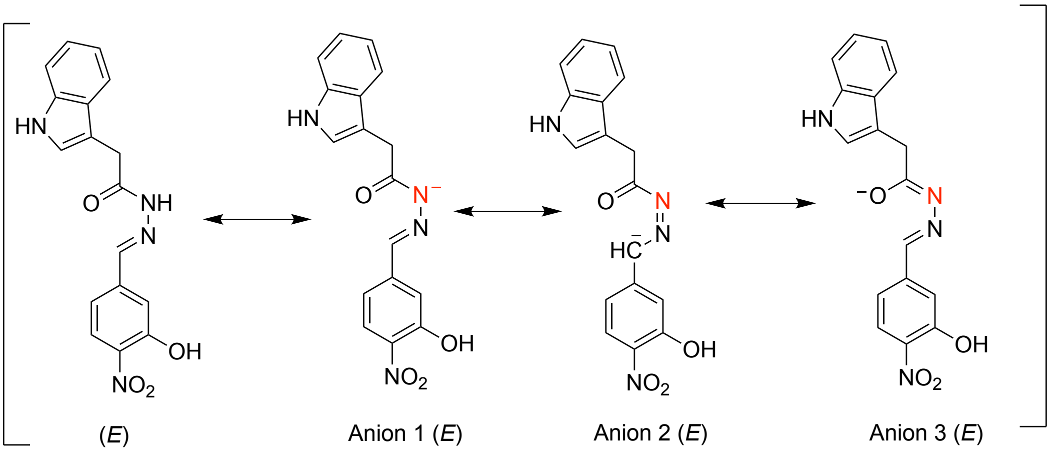

Supplementary Figure 19. Compound 3b *E* isomer and its corresponding anions.

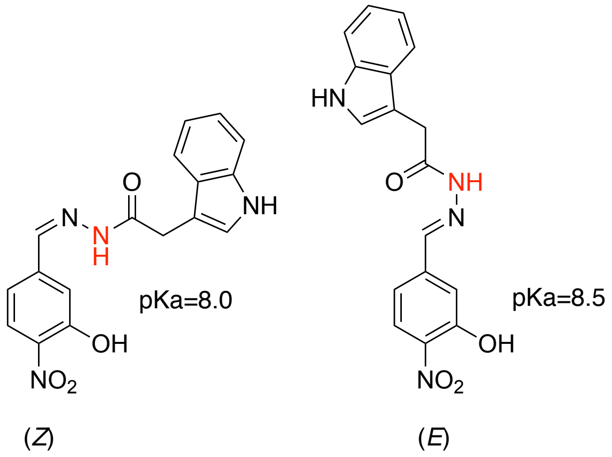

Supplementary Figure 20. Stereoisomers *Z* and *E* with their pKa values. After the geometry optimization, pKa prediction in aqueous media was performed for both stereoisomers by using Jaguar pKa prediction module (Figure 19).[3]

2. SUPPLEMENTARY TABLES

Supplementary Table 1. Area average and Relative peak area of the DCL compounds.

	Without Protein		With Protein	
Compounds	Area	RPA (%)	Area	RPA (%)
3c	301770	9.6	0	0.0
3a	856937	27.3	143022	18.8
3d	350202	11.1	63997	8.4
3e	1029897	32.8	331177	43.4
3b	603591	19.2	224555	29.4

Supplementary Table 2. Normalized relative peak area of the DCL compounds.

Compounds	Normalized Change of RPA
3c	-1,00
3a	-0,31
3d	-0,25
3e	0,32
3b	0,53

Supplementary Table 3. Measurement of acylhydrazones extinction coefficient ε.

Compound	λ_{max} (nm)	Data Fitting	ε ($L{\cdot}mol^{-1}{\cdot}cm^{-1}$)
3a	335	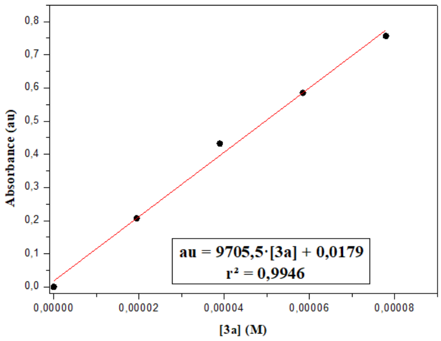	9705,5
3b	329	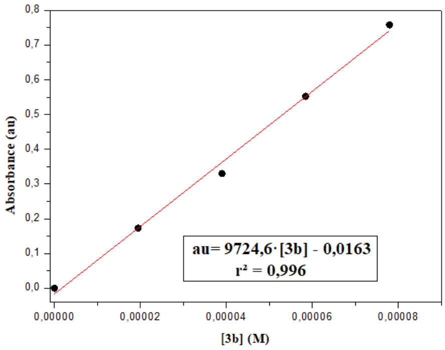	9724,6
3c	326	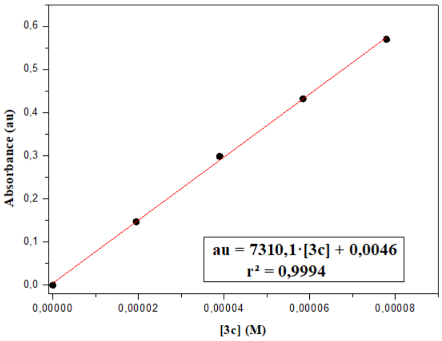	7310,1

3d	330	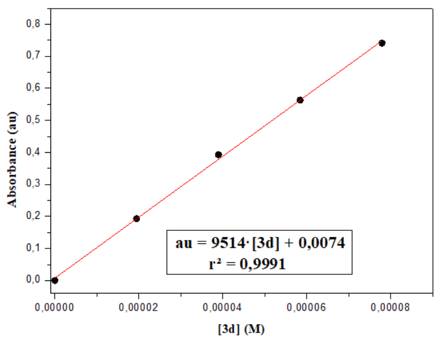	9514,0
3e	337	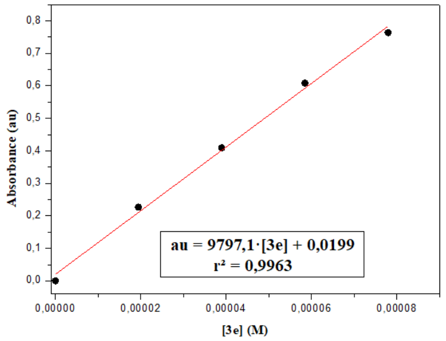	9797,1

Supplementary Table 4. Two significant ^{1}H-NMR shifts of intermediate I. They were calculated by MestReNova program to elucidate the intermediate structure.

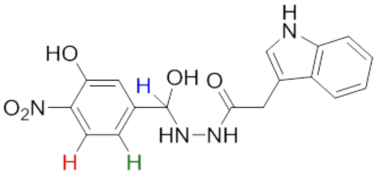

	^{1}H-NMR	
H	$\delta_{Experimental}$ (ppm)	$\delta_{Calculated}$ (ppm)
**H**	5,25	5,64
**H**	7,79	7,95
	^{13}C-NMR	
H	$\delta_{Experimental}$ (ppm)	$\delta_{Calculated}$ (ppm)
**H**	83,2	86
**H**	124,7	125,1

	^{1}H-NMR	
H	$\delta_{Experimental}$ (ppm)	$\delta_{Calculated}$ (ppm)
**H**	7,79	7,95
**H**	7,02	7,01

Supplementary Table 5. Permeability prediction in the PAMPA assay. Blood Brain Barrier assay for 10 commercial drugs used in the experiment validation and compounds with their predictive penetration in the CNS.[4] Data are the mean SD of 2 independent experiments. Source data are provided as a Source Data file.

	Bibl.	*Pe* (10^{-6} cm s^{-1})	Prediction
Atenolol	0.8	1.1 ± 0.7	
Caffeine	1.3	1.3 ± 0.6	
Desipramine	12	14.9 ± 0.9	
Enoxacin	0.9	1.4 ± 0.1	
Hydrocortisone	1.9	2.8 ± 0.8	
Ofloxacin	0.8	1.5 ± 0.4	
Piroxicam	2.5	1.3 ± 0.2	
Promazine	8.8	9.5 ± 0.6	
Testosterone	17	21.1 ± 0.9	
Verapamil	16	14.2 ± 0.1	
**3a**		1.7 ± 0.7	CNS -
**3b**		12.9 ± 0.8	CNS +
**3d**		2.5 ± 0.6	CNS +/CNS-

Supplementary Table 6. Calculated energies for stereoisomers *Z* and *E*. Energies calculated in hartree of compounds 3a-e. For compound 3b, their correspondent anions are also calculated (see Supplementary Figure 19 for structures). Energy difference (in kcal/mol) of stereoisomers *Z* and *E* (for calculated energies in water) is shown.

		**B3LYP/6-31G***		**HF/6-31G***	
		Water	Vacuum	Water	Vacuum
**3a**	***Z***	-1002.9244	-1002.8927	-997.1536	-997.1125
	E	-1002.9504	-1002.9280	-997.1743	-997.1455
**3b**	***Z***	-1176.0490	-1176.0198	-1169.0467	-1169.0091
	E	-1176.0613	-1176.0297	-1169.0563	-1169.0164
	**Anion 1 (*Z*)**	-1175.5668	-1175.4797	-1168.5521	-1168.4499
	**Anion 1 (*E*)**	-1175.5729	-1175.4868	-1168.5612	-1168.4578
	**Anion 2 (*Z*)**	-1175.0492	-1175.4794	-1168.0031	-1167.7445
	**Anion 2 (*E*)**	-1175.0552	-1174.8084	-1168.0102	-1167.7584
	**Anion 3 (*Z*)**	-1175.5635	-1175.4795	-1168.5512	-1168.4496
	**Anion 3 (*E*)**	-1175.5707	-1175.4871	-1168.5602	-1168.4581
**3c**	***Z***	-967.2448	-967.2107	-961.6327	-961.5918
	E	-967.2675	-967.2391	-961.6531	-961.6169
**3d (*R*)**	***Z***	-1119.6677	-1119.6434	-1113.1182	-1113.0079
	E	-1119.9686	-1119.6713	-1113.1393	-1113.1028
**3d (*S*)**	***Z***	-1119.6603	-1119.6429	-1113.1102	1113.0769
	E	-1119.6990	-1119.6728	-1113.1367	-1113.1025
**3e**	***Z***	-1325.9071	-1325.8755	-1319.8189	-1319.7780
	E	-1325.9302	-1325.9040	-1319.8364	-1319.8033

$\Delta E\ (E_Z - E_E)$

	**B3LYP/6-31G***	**HF/6-31G***
3a	16.3	13.0
3b	7.7	6.0
3c	14.2	12.8
3d (R)	19.4	13.2
3d (S)	24.3	16.6
3e	14.5	11.0

Supplementary Table 7. ADME descriptors for *Z* and *E* stereoisomers.

		logP(o/w)	logD	logS
3a	*E*	1.323	1.018	-3.516
	Z	1.395	1.018	-3.736
3b	*E*	2.581	2.038	-4.675
	Z	2.421	2.038	-4.183
3c	*E*	0.500	0.168	-2.709
	Z	1.076	0.168	-2.884
3d (*R*)	*E*	1.315	1,224	-3.912
	Z	1.821	1,224	-4.087
3d (*S*)	*E*	1.316	1,224	-3.912
	Z	1.913	1,224	-4.087
3e	*E*	1.855	1.872	-4.041
	Z	1.995	1.872	-4.216

Supplementary Table 8. Experimental log S of 18 commercial drugs.[5]

	**log S**
Benzocaine	-2.32
Aspirin	-1.72
Theophylline	-1.39
Antipyrine	-0.56
Atrazine	-3.85
Phenobarbital	-2.34
Diuron	-3.80
Nitrofurantoin	-3.47
Phenytoin	-3.99
Diazepam	-3.76
Testosterone	-4.09
Lindane	-4.64
Parathion	-4.66
Diazinon	-3.64
Phenolpthalein	-2.60
Malathion	-3.37
Chlorpyriphos	-5.49
Prostaglandin	-2.47

3. SUPPLEMENTARY METHODS

Kinetic experiments to determine the reaction order. The course of the reaction was studied for 7 hours. To determine the reaction order, the absorbance of aldehyde 1 and product 3b at increasing concentrations was measured in intervales of 15 minutes during two hours by HPLC (gradient from 15 to 85 % in 15 min). The kinetic experiment was performed adding the aldehyde 1 (1.2 μL, 50 mM, 1 eq., DMSO), the acylhydrazide 2b (3.6 μL, 1.5 M, 3 eq., DMSO), DMSO (28.8 μL) and buffer 20 mM TRIS, 0.5 M NaCl and 1mM $CaCl_2$, 1mM DTT pH 7.4 at 4 º C (5% DMSO). Asumming that the acylhydrazide's concentration keeps constant in all the experiment, data treatment was carried out with the aldehyde and the acylhydrazone concentration by fitting to the best model equation respect to the aldehyde. Absorbance area data were collected and treated by using a least squares algorithm to fit the equation for different reaction orders such as zero, first and second (Supplementary Equations 1, 2 and 3). Our experiments fit to pseudo-second order (Supplementary Figure 1).

Zero-order: $$[1] = [1]_0 - k' \cdot t \qquad (1)$$

First-order: $$ln[1] = ln[1]_0 - k' \cdot t \qquad (2)$$

Second-order: $$\frac{1}{[1]} = \frac{1}{[1]_0} + k' \cdot t \qquad (3)$$

Kinetic experiments for catalyst selection. Aldehyde 1 and product 3b absorbance was measured from 0 to 7 hours, and their concentrations were determined by a calibration curve equation. The reaction in presence of catalyst is finished after two hours. In order to investigate the K' value, aldehyde 1 and product 3b absorption and concentration were analyzed in intervales of 15 minutes during two hours by HPLC. The kinetic experiment was performed adding the aldehyde 1 (1.2 μL, 50 mM, 1 eq.), the acylhydrazide 2b (3.6 μL, 1.5 M, 3 eq.), the catayst or DMSO in the absence of catalyst (1.0 μL), DMSO (27.8 μL) and buffer 20 mM TRIS, 0.5 M NaCl and 1mM $CaCl_2$, 1mM DTT pH 7.4 (5% DMSO) at 4 º C. The experiments were fulfilled with three different catalysts: *p*-anisidine, *p*-phenilendiamine and aniline in two concentration: 15 mM and 50 mM. The data were collected and treated by using a least squares algorithm to fit the equation for pseudo-second order (Supplementary Figure 2).

Materials and equipment. All chemicals and solvents were used from commercial sources such as Sigma-Aldrich, Acros Organics, Alfa-Aesar, Fluorochem and TCI-Europe**.** Melting point was measured by Büchi Melting Point M-560 device. ^{1}H-NMR and ^{13}C-NMR spectra were collected by Bruker DPX 300MHz BACS-60 equipped with QNP 5mm sounding line, operated at 300 MHz and 75 MHz. Chemical shifts are reported in ppm (δ), using the correspondent deuterated solvent. Shifts multiplicity is s: single, d: double, m: multiplet, coupling constants values (J) are measured by Hz, acquired in the Nuclear Magnetics Resonance Centre of Complutense University of Madrid. Isomers shifts assignement was performed by Presch 2009 tables. Elemental analysis was performed by LECO CHNS-932 elemental analyzer of Universidad Complutense de Madrid. Reactions were purified by an automatic system Biotage chromatography (5 and 10 g. ZIP column). Dynamic combinatorial libraries and kinetics assays were analyzed by high-performance liquid chromatography- mass spectroscopy (HPLC-MS), in HPLC Surveyor and a Thermo Mod. FinniganTM LXQ TM Ion trap mass spectrometry system (Thermo Mod. FinniganTM LXQ TM). Solvents and formic acid were acquired from J.T. Baker and Sigma-Aldrich respectively. Analyses were performed using a reversed phase HPLC column (ACE Excel 3 C18-PFP 4.6 x 100 mm, 3 µm), using an injection volume of 25 µL, a flow rate of 1 mL/min and a gradient (15-85%) in 15 min of acetonitrile in water, both containing 0.1 % formic acid at 25 ºC. Positive ion mass spectra were acquired using electrospray ionization (drying temperature 300 ºC, sheath gas flow 60, HV capillary 6000 V, source voltage 5.50 kV, source current 100 µA).

General synthesis of acylhydrazones. Over a solution of 3-hydroxy-4-nitrobenzaldehyde (1.2 eq.) in solvent MeOH or EtOH (60 mL), selected acylhydrazide (1eq.) is added. The reaction mixture was stirred at reflux 10-16 h. The purification of the crude is by recrystallization or by chromatography on an automatic silica gel column Biotage system using as eluents DCM / MeOH. It yielded the desired compounds (40-90 % yields) as colorful solids, with different proportions of isomers E/Z. The E/Z ratio was determined by ^{1}H-NMR analysis (see Supplementary Figure 3 and Supplementary Discussion).

Calculation of molar extinction coefficient. Absorbance was measured at four concentrations for acylhydrazones 3a-e. Linear regression of the absorbance values per concentration was performed to derive the extinction coefficient ε. Beer-Lambert law:

the length of the pass light (L) is 1cm (see Supplementary Table 3 and Supplementary Discussion).

Fluorescence emission experiments. Tryptophan emission fluorescence of *d*NCS-1 (0.8 μM) was measured at increasing concentrations of ligands 3(a-d) to determine their affinity and calculate the apparent dissociation constant, K_d. Fluorescence measurements were performed by Jobin Yvon Floromax4 spectrofluorimeter equipped with Peltier thermostat in buffer 50 mM Tris, pH 7.9, 125 mM NaCl, 0.5 mM $CaCl_2$ and 5% DMSO at 5 º C. The excitation wavelength was fixed at 295 nm, and the emission spectra range were collected over 300-400 nm. Firstly, ligands were verified not to emit in the emission range of interest. Then, three independent experiments were performed for each ligand increasing *d*NCS1:ligand molar ratio from 1:0 to 1:120 equivalents. Fluorescence intensities were normalized and represented as $(I_0 - I)/I_0$. I stands for the observed fluorescence emission at 345 nm (fluorescence maximum), and I_0 is the observed fluorescence emission of the protein itself without any small molecule. The apparent dissociation constant was obtained using a least squares algorithm to fit the experimental data to a 1:1 stoichiometry model.[6] The fitting was performed with KaleidaGraph Data Analysis Program.

Protein stability experiments. To monitor the tolerance of *d*NCS-1 to DMSO, Trp emission fluorescent spectra were recorded at increasing concentrations of DMSO and under the same experimental conditions described above. Tryptophan residues are particularly valuable probes since the indole ring is very sensitive to its environment. When a protein is unfolding, and due to the progresive exposure of tryptophans to the solvent, the emission maxima (λ_{max}) of the protein spectrum shifts to 350nm.[7,8] The presented data (Figure 14) indicate that *d*NCS-1 tolerates up to 20% DMSO since no shift is observed at those concentrations. In fact, at 40% DMSO the protein is mainly folded since a small shift (1nm) is observed. Therefore, the DCC has been performed in conditions where the protein is properly folded. The ability of this protein to bind Ca^{2+} confers stability to the fold and this might be the reason of the tolerance to DMSO. (see Supplementary Figure 15 and 16).

Protein expression and purification. *Drosophila* and human NCS-1 were over-expressed in *E. coli* and subsequently purified to homogeneity as previously described.[9]

In brief, the Ca^{2+} bound proteins were subjected to an hydrophobic chromatography followed by an anionic exchange.

Diffraction data collection and structure solution. Purified *h*NCS-1 was dialyzed against buffer 20mM sodium acetate pH 6.5, 0.5mM $CaCl_2$, 0.5mM DTT and concentrated to 10mg/ml. Crystals of the ligand-free protein were obtained at 277 K, using sitting drop vapour diffusion techniques and the crystallization solutions reported previously.[1] In summary, thick plates grew when mixing 1 μL of concentrated protein with 1 μL of reservoir solution containing 0.1 M sodium cacodylate trihydrate pH 6.5, 0.2 M sodium acetate trihydrate, 30% (v/v) polyethylene glycol 8000 (solution 28, Crystal Screen I, Hampton Research). To obtain crystals of hNCS-1 bound to 3b, native crystals were soaked during 16-20h in the crystallization solution additionally containing 10mM 3b and 3% (v/v) DMSO. To cryoprotect the crystals, 10% (v/v) ethylene glycol was added to the soaking solution. Then, crystals were flash-frozen in N_2 (l). Diffraction data of a *h*NCS-1/3b crystals were collected at 100 K and 0.978979 Å wavelength at the ALBA synchrotron radiation source (BL13 beamline) and processed with XDS[10] and Aimless.[11] The structure of the complex was solved with Phaser,[12] using as search model the structure of *h*NCS-1 (PDB code 1g8i). The 3b dictionary containing geometrical restraints was generated with Grade Web Server (http://grade.globalphasing.org) and further improved with data extracted from the Cambridge Structural Database.[13] Refinement map and model validation calculations were performed with Phenix.[14,15,16] The Ramachandran plot statistics of the final model showed 99.46% residues in favoured regions and 0.0% outliers. Details on data processing and refinement are shown in Table 1. Analysis of the structure was performed with programs from CCP4 package.[17] Images were prepared with Pymol.[3]

Parallel artificial membrane permeability assays. Prediction of the brain penetration was evaluated using a parallel artificial membrane permeability assay (PAMPA).[4] Ten commercial drugs, phosphate buffer saline solution at pH 7.4 (PBS). DMSO and dodecane were purchased from Sigma, Across organics, Aldrich and Fluka. The porcine polar brain lipid (PBL) (catalog no. 141101) was from Avanti Polar Lipids. The donor plate was a 96-well filtrate plate (Multiscreen® IP Sterile Plate PDVF membrane, pore size is 0.45 μM, catalog no. MAIPS4510) and the acceptor plate was an indented 96-well plate (Multiscreen®, catalog no. MAMCS9610) both from Millipore. Filter PDVF

membrane units (diameter 30 mm, pore size 0.45 μm) from Symta were used to filter the samples. A 96-well plate UV reader (Thermoscientific, Multiskan spectrum) was used for the UV measurements. Test compounds: 3-5 mg of Cafeine, Enoxacine, Hydrocortisone, Desipramine, Ofloxacine, Piroxicam, Testosterone, 12 mg of Promazine and 25 mg of Verapamile and Atenolol, were dissolved in DMSO (250 μL). 25 μL of these stock solutions were taken and 225 μL of DMSO and 4750 μL of PBS pH=7.4 buffer were added to reach 5% of DMSO concentration in the experiment. These solutions were filtered. The acceptor 96-well microplate was filled with 180 μL of PBS/DMSO (95/5). The donor 96-well plate was coated with 4 μL of porcine brain lipid in dodecane (20 mg mL-1) and after 5 min, 180 μL of each compound solution was added. 0.20 mg of compound **3b** was dissolved in 250 μL of DMSO and 4750 μL of PBS pH=7.4 buffer and then added to the donor 96-well plate. Then the donor plate was carefully placed on the acceptor plate as a "sandwich", which was left undisturbed for 2h and 30 min at 25 °C. During this time compounds diffused from the donor plate through the brain lipid membrane into the acceptor plate. After incubation, the donor plate was removed. In the acceptor and donor wells UV plate reader determined the concentrations. Every sample was analyzed at three to five wavelengths, in three wells and in two independent runs. Results are given as the mean [standard deviation (SD)] and the average of the two runs is reported. Ten quality control compounds (previously mentioned) of known BBB permeability were included in each experiment to validate the analysis set (Table 4).[2] Finally, a good correlation between experimental-described values was obtained *Pe* (exp.) = 1.094 (bibl.) + 0.1264 (R^2= 0.9531). From this equation and following the pattern established in the literature for BBB permeation prediction[1] we could classify compounds as CNS + when they present a permeability > 4.50 x 10^{-6} cm s^{-1} (see Supplementary Figure 18 and Supplementary Table 6).[4]

Stereoisomers energy calculations and ADME descriptors. Geometry optimization and energy calculation (Supplementary Figure 19 and 20, Supplementary Table 7) for both *Z/E* stereoisomers of compounds **3a-e**, and the corresponding anions (conjugated bases) of compound **3b** were performed by using the quantum mechanics calculation software Jaguar[18] with DFT B3LYP/6-31G* and R-HF (6-31G*) methods, in water and in vacuum. Furthermore, ADME descriptors have been calculated (Supplementary Table 8) by means of QikProp software[19] (log P and log S), and Chemicalize software[20] (log D). In Supplementary Table 9 the log S of 18 commercial drugs can be found.

4. SUPPLEMENTARY DISCUSSION

Characterization of *E-N*'-(3-hydroxy-4-nitrobenzylidene)furan-2-carbahydrazide (3a):

3-hydroxy-4-nitrobenzaldehyde (319 mg, 1.91 mmol), 2-furoic acid hydrazide (200 mg, 1.59 mmol), MeOH (60 mL). The product was filtered and washed with MeOH to afford as a yellow solid (0.30 g, 70%) (E > 99). m.p: 256 - 257 ºC. ^{1}H-RMN (300 MHz, DMSO-d$_6$) δ 12.07 (s, 1H), 11.18 (s, 1H), 8.40 (s, 1H), 7.98 (m, 2H), 7.49 (d, J = 1.7 Hz, 1H), 7.40 – 7.26 (m, 2H), 6.72 (d, 1H, J = 1.7 Hz). ^{13}C-RMN (75 MHz, DMSO-d$_6$): δ 164.5, 152.3, 146.7, 145.2, 140.5, 140.0, 137.2, 125.9, 118.3, 118.5, 116.8, 112.6. HRMS (m/z): [M]$^+$ calcd. for $C_{12}H_9N_3O_5$, 275.0545; found, 275.0542; Anal. Calcd, for $C_{12}H_9N_3O_5$: C, 52.37%; H, 3.30%; N, 15.27%. Found: C, 52.43%; H, 3.30%; N, 15.11%. HPLC-MS: t_R: 7.02 min, [M+H]$^+$ = 276 m/z.

Characterization of *(E/Z)-N'*-(3-hydroxy-4-nitrobenzylidene)-2-(1H-indol-3-yl)acetohydrazide (3b):

3-hydroxy-4-nitrobenzaldehyde (330 mg, 1.97 mmol), indole-3-acetic hydrazide (300 mg, 1.58 mmol), MeOH (60 mL). The product was filtered and washed with MeOH to afford as an orange solid (0.30 g, 70%) (E/Z = 40:60). m.p: 216 – 217 ºC. ^{1}H-RMN (300 MHz, DMSO-d$_6$) δ 11.79 (s, 1H, E), 11.54 (s, 1H, Z), 11.19 (s, 1H), 7.88 (s, 1H), 7.70-7.65 (m, 1H), 7.39-7.36 (m, 1H), 7.27-7.25 (m, 1H), 7.15(s, 1H), 7.02-6.98 (m, 1H), 6.92-6.88 (m, 1H), 6.78 (s, 1H), 6.72-6.68 (m, 1H), 5.89 (s, 1H), 3.68 (s, 2H). ^{13}C-RMN (75 MHz, DMSO-*d$_6$*): δ 172.1, 150.8, 148.1, 142.8, 137.4, 136.4, 130.5, 125.9, 123.9, 122.6, 121.0, 120.2, 119.2, 116.7, 111.9, 110.6, 42.5. HRMS (m/z): [M]$^+$ calcd. for $C_{17}H_{14}N_4O_4$, 338.1027; found, 338.1015; Anal. Calcd, for $C_{17}H_{14}N_4O_4$: C, 60.35 %; H, 4.17 %; N, 16.56 %. Found: C, 60.13 %; H, 4.20 %; N, 16.56 %. HPLC-MS: t_R: 8.45 min, [M+H]$^+$ = 339 m/z.

Characterization of *(E)-N'-(*3-hydroxy-4-nitrobenzilidene)-3-metoxypropanehydrazide (3c):

3-hydroxy-4-nitrobenzaldehyde (339 mg, 2.00 mmol), 3-metoxypropionic acid hydrazide (180 µL, 1.69 mmol), MeOH (60 mL). It was chromatographed on automatic silica gel column Biotage system using DCM / MeOH (0- 6 %) as eluents. It was a

yellow solid (0.44 g., 97 %) (E > 99). m.p: 163 - 164ºC. ^{1}H-NMR (300 MHz, DMSO-*d$_6$*): δ 11.61 (s, 1H, E), 11.13 (s, 2H), 8.13 (s, 1H), 7.95-7.92 (m, 3H), 3.66-3.58 (m, 2H), 3.24 (s, 3H), 2.47 (t, 2H, *J* = 6.8 *Hz*). ^{13}C-NMR (75 MHz, DMSO-*d$_6$*): δ 172.1, 151.8, 143.1, 140.2, 139.8, 136.6, 125.3, 117.4, 67.4, 57.4, 34.4. HRMS (m/z): $[M]^+$ calcd. for $C_{11}H_{13}N_3O_5$, 267.08593; found, 267.08552; Anal. Calcd, for $C_{11}H_{13}N_3O_5$: C, 49.44 %; H, 4.90 %; N, 15.72 %. Found: C, 49.51 %; H, 4.82 %; N, 15.61 %. HPLC-MS: t_R: 6.47 min, $[M+H]^+$ = 268 m/z.

Characterization of (E/Z)-2-hydroxy-*N*'-(3-hydroxy-4-nitrobenzilidene)-2-phenylacetohydrazide (3d):

3-hydroxy-4-nitrobenzaldehyde (241 mg, 1.44 mmol), mandelic acid hydrazide (201 mg, 1.21 mmol), MeOH (20 mL). The crude was chromatographed on automatic silica gel column Biotage system using DCM / MeOH (3 – 6%) to obtain a yellow solid (0.18 g, 47%) (E/Z = 76:24). m.p: 199 - 200 º C. ^{1}H-NMR (300 MHz, DMSO-*d$_6$*): δ 11.71 (s, 1H, E), 11.64 (s, 1H, Z), 11.18 (s, 1H), 8.44 (s, 1H), 8.00 – 7.86 (m, 1H), 7.56 – 7.45 (m, 1H), 7.50 (s, 1H), 7.48 – 7.19 (m, 5H), 6.44 (d, *J* = 4.4 Hz, 1H), 5.14 (d, *J* = 4.4 Hz, 1H).^{13}C-NMR (75 MHz, DMSO-*d$_6$*): δ 173.9, 168.7, 152.3, 145.7, 141.2, 140.5, 140.4, 137.2, 127.8 (2C), 126.8 (2C), 117.9, 116.3, 73.44. HRMS (m/z): $[M]^+$ calcd. for $C_{15}H_{13}N_3O_5$, 315.08676; found, 315.08552; Anal. Calcd, for $C_{15}H_{13}N_3O_5$: C, 57.14 %; H, 4.16 %; N, 13.33 %. Found: C, 56.27 %; H, 4.13 %; N, 12.97 %. HPLC-MS: t_R: 7.38 min, $[M+H]^+$ = 316 m/z.

Characterization of *(E)-N*'-(3-hydroxy-4-nitrobenzilidene)thiophene-2-carbohydrazide (3e):

3-hydroxy-4-nitrobenzaldehyde (282 mg, 1.69 mmol), 2-thiophenecarboxylic acid hydrazide (200 mg, 1.41 mmol), EtOH (60 mL). The product was filtered and washed with EtOH to provide a yellow solid (0.29 g, 70%) (E > 99). m.p: 250-251ºC. ^{1}H-NMR (300 MHz, DMSO-*d$_6$*): δ 12.06 (s, 1H), 11.21 (s, 1H), 8.39 (s, 1H), 8.06 (s, 1H), 7.99-7.97 (m, 2H), 7.52 (s, 1H), 7.34 (s, 1H), 7.27-7.24 (m, 1H). ^{13}C-NMR (75 MHz, DMSO-*d$_6$*): δ 160.0, 151.8, 146.3, 145.9, 145.0, 139.8, 136.6, 128.0, 126.5, 125.4, 118.4, 117.1. HRMS (m/z): $[M]^+$ calcd. for $C_{12}H_9N_3O_3S$, 291.03136; found, 291.03138. Anal. Calcd, for $C_{12}H_9N_3O_3S$: C, 49.48 %; H, 3.11 %; N, 14.43 %; S, 11.01%. Found: C, 49.71 %; H, 3.10 %; N, 14.39 %; S, 11.05 %. HPLC-MS: t_R: 8.28 min, $[M+H]^+$ = 292 m/z.

Calculation of Relative Peak Area and Normalized RPA.

For quantifying the influence of the protein template, the Relative Peak Area (RPA) data was normalized (Supplementary Equation 4; Supplementary Table 1 and 2, Supplementary Figure 9 and 10). Positive bars indicate acylhydrazones amplification due to template effect, while negative bars indicate reduction. The product distribution was established using independently prepared reference compounds and quantified with each peak area being corrected by the molar extinction coefficient (ε) of the corresponding acylhydrazone (Supplementary Table 3).

$$\text{RPA Normalized Variation} = \frac{\text{RPA}_{\text{with prot}} - \text{RPA}_{\text{without prot.}}}{\text{RPA}_{\text{without prot.}}} \quad (4)$$

5. SUPPLEMENTARY REFERENCES

1. Bourne, Y., J. Dannenberg, Pollmann, V., Marchot, P., Pongs, O. Immunocytochemical localization and crystal structure of human frequenin (neuronal calcium sensor 1). *J. Biol. Chem.* **276**(15): 11949 (2001).
2. Crivori, P.; Cruciani, G.; Testa, B. Predicting Blood-Brain Barrier Permeation from Three-Dimensional Molecular Structure. *J. Med. Chem.* **43**, 2204 (2000).
3. The PyMOL Molecular Graphics System, V. S., LLC. (2013).
4. Di, L.; kerns, E. H.; Fan, K.; McConnell, O. J.; Carter, G. T. High throughput artificial membrane permeability assay for blood–brain barrier *Eur. J. Med. Chem.* **38**, 223 (2003).
5. Hou, T. J., Xia, K., Zhang, W., Xu, X. J., ADME Evaluation in Drug Discovery. 4. Prediction of Aqueous Solubility Based on Atom Contribution Approach. *J. Chem. Inform. Computer Sciences* 44, 266-275 (**2004**).
6. Mansilla, A., Chaves-Sanjuan, A., Campillo, N. E., Semelidou, O., Martínez-González, L., Infantes, L., González-Rubio, J. M., Gil, C., Conde, S., Skoulakis, E. M. C., Ferrús, A., Martínez, A., Sánchez-Barrena, M. J. Interference of the complex between NCS-1 and Ric8a with phenothiazines regulates synaptic function and is an approach for fragile X syndrome *Proc. Natl. Acad. Sci. USA* **114***,* E999 (2017).
7. Eftink, M. R. The use of fluorescence methods to monitor unfolding transitions in proteins. *Biophys. J.* **66**, 482-501 (1994).
8. Duy, C., Fitter, J. How aggregation and conformational scrambling of unfolded states govern fluorescence emission spectra. Biophys. J. 90, 3704-3711 (2006).
9. Baños-Mateo, S., Chaves-Sanjuán, A., Mansilla, A., Ferrús, A., Sánchez-Barrena, M. J. Frq2 from *Drosophila melanogaster*: cloning, expression, purification, crystallization and preliminary X-ray analysis. *Acta Cryst*. **70**, 530 (2014).
10. Kabsch, W. Xds. Acta Crystallogr. D. Biol. Crystallogr. **66**, 125 (2010).
11. Evans, P. R., Murshudov, N. G. How good are my data and what is the resolution? *Acta Crystallogr. D. Biol. Crystallogr*. **69**, 1204 (2013).
12. McCoy, A. J., Grosse-Kunstleve, R. W., Adams, P. D., Winn, M. D., Storoni L. C., Read, R. J. Phaser crystallographic software. *J. Appl. Crystallogr*. **40**, 658 (2007).
13. Groom, C. R., I. J. Bruno, et al. , Lightfoot, M. P., Ward, S. C. The Cambridge Structural Database. *Acta Crystallogr. B. Struct. Sci. Cryst. Eng. Mater.* **72,** 171 (2016).
14. Adams, P. D., Afonine, P. V., Bunkóczi, G., Chen, V. B., Davis, I. W., Echols, N., Headd, J. J., Hung, L.-W., Kapral, G. J., Grosse-Kunstleve, R. W., McCoy, A. J., Moriarty, N. W., Oeffner, R., Read, R. J., Richardson, D. C., Richardson, J. S., Terwilliger, T. C., and Zwart, P. H. PHENIX: a comprehensive Python-based system for macromolecular structure solution. *Acta Crystallogr. D. Biol. Crystallogr.* **66**, 213 (2010).
15. Afonine, P. V. *et al.* FEM: a feature-enhanced map. *Acta Crystallogr. D. Biol. Crystallogr.* **71**, 646-666 (2015)
16. Liebschner, D *et al.* Polder maps: improving OMIT maps by excluding bulk solvent. *Acta Crystallogr.* **D73**, 148-157 (2017).

17. Winn, M. D., Ballard, C.C., Cowtan, K. D., Dodson, E. J., Emsley, P., Evans, P. R., Keegan, R. M., Krissinel, E. B., Leslie, A. G., McCoy, A., McNicholas, S. J., Murshudov, G. N., Pannu, N. S., Potterton, E. A., Powell, H. R., Read, R. J., Vagin, A., Wilson, K. S. Overview of the CCP4 suite and current developments." *Acta Crystallogr. D. Biol. Crystallogr.* **67**, 235 (2011).
18. Bochevarov, A. D., Harder, E., Hughes, T. F., Greenwood, J. R., Braden, D. A., Philipp, D. M., Rinaldo, D., Halls, M. D., Zhang, J., Friesner, R. A. Jaguar: A high-performance quantum chemistry software program with strengths in life and materials sciences. *Int. J. Quantum Chem.* **113**, 2110-2142 (2013).
19. Schrödinger Release 2018-4: QikProp, Schrödinger, LLC, New York, NY, 2018
20. https://chemaxon.com/products/chemicalize
