# Supplementary material for: Insights into real-time chemical processes in a calcium sensor protein-directed dynamic library
Source: Nat Commun. 2019 Jun 26;10:2798. doi: 10.1038/s41467-019-10627-w (PMC6595003; doi:10.1038/s41467-019-10627-w)
Supplement: Supplementary file 3 — Description of Additional Supplementary Files [file 41467_2019_10627_MOESM3_ESM.pdf]

Description of Additional Supplementary Movies

File name: Supplementary Movie 1

Description: Video recording the 2DUF-TOCSY experiments monitoring the catalytic pathway. The experiment started with the injection into the NMR tube using a fast mixing device of 2b to the solution of 1 and *p*-anisidine.
